# At least 10 minutes of seated rest is required for stabilisation of blood-based hydration biomarkers

**DOI:** 10.1007/s00421-025-06048-x

**Published:** 2025-11-21

**Authors:** Kirsty M. Reynolds, Samantha N. Rowland, Mark P. Funnell, Tom Clifford, Liam M. Heaney, Stephen A. Mears, Stephen J. Bailey, Lewis J. James

**Affiliations:** 1 School of Sport, Exercise and Health Sciences, Loughborough University, Loughborough, UK; 2 NIHR Applied Research Collaboration East Midlands, Diabetes Research Centre, University of Leicester, Leicester, UK; 3 NIHR Leicester Biomedical Research Centre, University Hospitals of Leicester NHS Trust and University of Leicester, Leicester, UK

**Keywords:** Postural control, Blood volume, Fluid balance, Blood sampling, Plasma, Haemodynamic

## Abstract

Postural changes elicit well-described haemodynamic effects on plasma volume, affecting blood-based hydration variables. While the time required for plasma volume stabilisation is well-established for supine rest, less is known for blood samples collected in a seated position, as is commonplace in physiology and nutrition experiments. Seventeen healthy participants (9 males; 8 females) stood stationary for 20 min in an upright position before walking on a treadmill at 4 km/h for 20 min (to simulate active travel to blood sampling locations). After walking, participants sat upright (within 7 ± 1 s), and venous blood samples were drawn from a cannula at 0, 5, 10, 20, 30 and 40 min after sitting. Time points were compared to 40 min. At 0 and 5 min, blood (0 min: -3.3%; *P* < 0.001; 5 min: -1.2%; *P* = 0.034) and plasma (0 min: -5.9%; *P* < 0.001; 5 min: -1.7%; *P* = 0.035) volume were lower than 40 min values. Conversely, plasma osmolality was higher at 0 min (+ 0.61%; *P* = 0.005) and 5 min (+ 0.55%; *P* = 0.007), whilst blood/plasma volume and plasma osmolality were not different from 10 min onwards. Plasma sodium, potassium and chloride concentrations were not different from 40 min throughout the sitting period (*P* ≥ 0.056). These data demonstrate that blood/plasma volume, as well as plasma osmolality were affected by posture and/or walking, but all are not significantly different from 40 min within 10 min of seated rest. Therefore, studies involving blood-based hydration measures taken in an upright seated position should allow at least 10 min of postural control prior to sampling.

**Supplementary Information:**

The online version contains supplementary material available at 10.1007/s00421-025-06048-x.

## Introduction

Blood-based hydration biomarkers, including plasma volume, osmolality and electrolyte concentrations, are important outcomes in various physiological studies, particularly for hydration and environmental physiology research. It has long been recognised that changes in posture have haemodynamic effects that influence blood and plasma volume, mainly due to changes in hydrostatic pressure caused by posture change (Thompson et al. 1928; Fawcett and Wynn 1960; Hagan et al. 1978; Harrison 1985; Shirreffs and Maughan 1994; Kargotich et al. 1998; Lippi et al. 2015).

Changes in blood/plasma volume may also influence other blood-based measures and potentially subsequent physiological responses (e.g., cardiovascular outcomes such as blood pressure, stroke volume and cardiac output); therefore, resolving the time required for stabilisation of blood/plasma volume is vital to ensure measurement reliability. Assessment of total haemoglobin mass through carbon monoxide rebreathing includes a 20 min seated rest prior to sampling (Siebenmann et al. 2017) as per Cores Of Reproducibility in Physiology protocols. Since haemoglobin is measured in estimates of change in plasma volume there may be implications for accuracy if samples are taken without adequate prior seated rest. Blood samples in research settings are routinely taken in supine, semi-supine and seated positions, with it being common for participants to have actively travelled (e.g., walked) very shortly before the sample is taken (i.e., to reach the location of blood sampling). Movement from a standing to rested position leads to increased blood/plasma volume, with stabilisation in a supine position occurring within 30 min (Shirreffs and Maughan 1994; Lippi et al. 2015). However, seated rest represents a smaller shift in body position, meaning the time required for blood/plasma volume stabilisation may be less, potentially reducing the time burden for participants and researchers in studies, whilst preserving accuracy of hydration assessment.

This study examined the time required from walking to sitting in an upright seated position for stabilisation of blood/plasma volume, as well as other blood-based hydration biomarkers following transition from a standing posture. As exercise, even low-intensity exercise, further decreases plasma volume (James et al. 2017; Liddle et al. 2018), we incorporated a 20 min treadmill walk prior to sitting to simulate active travel to the laboratory and induce larger postural change with sitting. We hypothesised that plasma volume stabilisation with upright sitting following simulated active travel (i.e. low-intensity exercise) would occur in less than 30 min, as previously observed when lying supine.

## Methods

### Participants

 Seventeen healthy individuals (9 males, 8 females) completed the study (mean ± standard deviation) age (26 ± 4 y), height (1.74 ± 0.12 m), body mass (74.4 ± 15.3 kg). Three females were regularly menstruating (self-reported), and 5 females were hormonal contraceptive users (*n* = 2 progesterone only pill users, *n* = 2 monophasic combined pill users, and *n* = 1 vaginal ring). Menstrual cycle phase and contraceptive type/phase were not controlled between participants since we were interested in acute changes, and the study consisted of a single visit. The study gained ethical approval from Loughborough University Ethics (Human Participants) Sub-Committee (Ethics Code: LEON 8071), with participants providing written informed consent and completing a health screening questionnaire before participation.

### Protocol

Participants arrived at the laboratory after an overnight fast (food and fluid) and having avoided exercise that morning. All experimental trials were completed in temperate conditions (25.1 °C ± 0.1 °C; 46.6%RH ± 0.6%RH). A flexible 20-gauge cannula was inserted into an antecubital vein for subsequent blood sampling. Participants then stood still for 20 min, before walking on a treadmill (4 km/h) for 20 min. After walking, participants assumed an upright seated position (within 7 ± 1 s), with blood samples (8 mL) drawn 0, 5, 10, 20, 30 and 40 min after sitting. For each sample, 2 mL of blood was drawn and discarded, with a further 8 mL drawn into a separate sterile syringe and used for analyses. The cannula was flushed with ~ 8 mL sterile saline after sampling.

### Sample processing and analysis

 At each time point, blood was dispensed into 3 × 1 mL tubes (one lithium heparin, two containing K2 EDTA) and a pre-chilled 5 mL lithium heparin tube before immediate centrifugation (3500 g, 10 min, 4 °C). Plasma was then aliquoted and stored at -80 °C until analysis. The cyanmethemoglobin method (in duplicate; CV 0.7%) and microcentrifugation (in triplicate; CV 0.5%) were used to determine haemoglobin and haematocrit, respectively, from EDTA treated blood to estimate changes in blood, red cell, and plasma volume, relative to 40 min (Dill and Costill 1974). 40 min was chosen as the baseline based on previous data where plasma volume stabilised after 30 min from standing to lying (Shirreffs and Maughan 1994), therefore, ensuring sufficient time to reach a plateau and providing a stable comparator for earlier time points. Lithium heparin treated blood was used to determine plasma osmolality via freezing point depression in duplicate (Gonotec Osmomat 030 Cryoscopic Osmometer; Gonotec; CV 0.26%). Plasma sodium and potassium concentrations were analysed by flame photometry in duplicate (M410C Flame Photometer, Sherwood Ltd., Cambridge, UK; CV 1.3% and 1.6%, respectively) and plasma chloride concentration was analysed by coulometric titration (Sherwood Scientific 926 S Chloride meter, Sherwood Scientific Ltd., Cambridge, UK; CV 0.5%) using lithium heparin treated blood.

### Statistical analyses

 Statistical analyses were completed using Statistical Package for Social Sciences (SPSS version 27; Chicago, IL, USA). Normality of data sets and paired differences were assessed using Shapiro-Wilk tests (*P* > 0.05). All data was normally distributed except plasma volume. Statistical significance was set at *P* ≤ 0.05 and results were presented as mean ± SD for normally distributed data or median ± interquartile range (IQR) for non-normally distributed data. Sex differences were analysed using two-way mixed ANOVAs. Data were analysed using one-way repeated measures ANOVAs, followed by paired t-tests or Wilcoxon signed rank tests (plasma volume data), as appropriate. The purpose of the study was to identify time points when valid measurements can be taken following stable posture (i.e., not introduce error related to postural artefacts), we chose to take the least conservative statistical approach to minimise type 1 error risk. Therefore, where violation of sphericity was present for ANOVA, the degrees of freedom were corrected using Huynh-Felt and for post-hoc analyses, no correction for multiple comparisons was used. Where statistically significant differences from 40 min were identified, 95% confidence intervals (CI) of the mean difference or median paired differences are presented.

## Results

### Blood and plasma volume

**Fig. 1:**
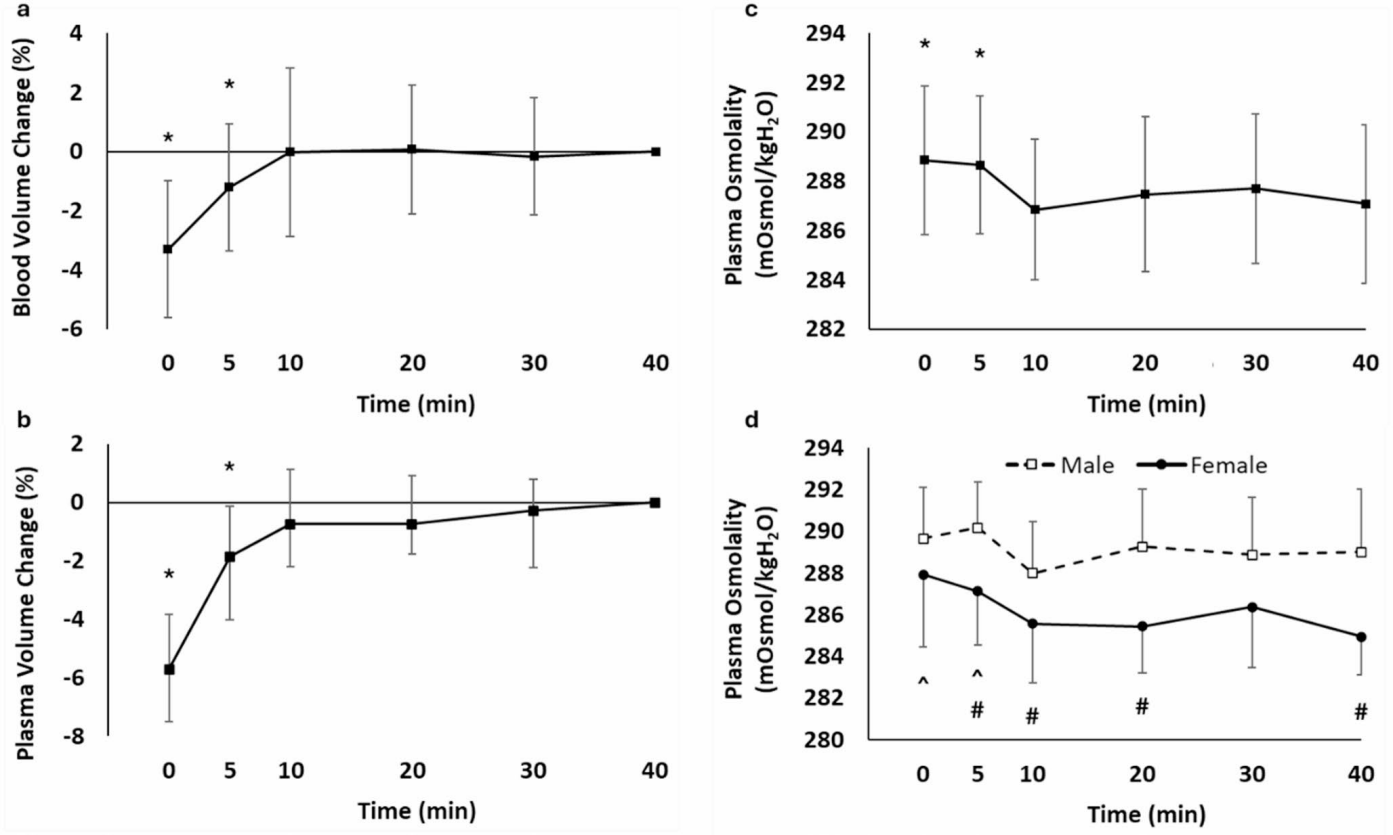
Blood volume change (%; **a**), plasma volume change (%; **b**), plasma osmolality (mOsmo/kgH2O; **c**), and plasma osmolality for males and females (mOsmo/kgH2O; **d**) immediately upon sitting (0 min) to 40 min. * = significantly different from 40 min; # = significantly different between males and females; ^ = in females only time point is significantly different from 40 min. Presented data are mean ± SD except plasma volume which are median ± IQR

There were main effects for blood (*P* < 0.001) and plasma (*P* < 0.001) volume (Fig. 1a and b). Compared to 40 min, blood volume was decreased at 0 min (-3.3%; 95% CI -4.4 to -2.2%; *P* < 0.001) and 5 min (-1.2%; 95% CI -2.22 to -0.19%; *P* = 0.034) but was not different at any other time point (*P* ≥ 0.742). Similarly, compared to 40 min, plasma volume was decreased at 0 min (-5.9%; 95% CI -7.5 to -3.82%; *P* < 0.001) and 5 min (-1.7%; 95% CI -4.0 to -0.14%; *P* = 0.035), but was not different at any other time point (*P* ≥ 0.600). Individual participant data are presented in Supplementary Fig. 1a/b. There were no sex differences in plasma volume (time x sex interaction *P* = 0.492).

### Plasma osmolality

There was a main effect for plasma osmolality (*P* < 0.001; Fig. 1c), which compared to 40 min was increased at 0 min (+ 2 mOsmol/kgH2O; 95% CI 1 to 3 mOsmol/kgH2O; *P* = 0.005) and 5 min (+ 1 mOsmol/kgH2O; 95% CI 0 to 2 mOsmol/kgH2O; *P* = 0.007), with no other differences (*P* ≥ 0.132). Individual participant data are presented in Supplementary Fig. 1c. Plasma osmolality displayed a main effect of sex (*P* < 0.001; Fig. 1d), as well as a time x sex interaction (*P* = 0.031). In males, plasma osmolality was not different compared to 40 min at any other time point (*P* ≥ 0.069), but in females was greater at 0 min (+ 3 mOsmol/kgH2O; 95% CI -2 to + 2 mOsmol/kgH2O; *P* = 0.009) and 5 min (+ 2 mOsmol/kgH2O; 95% CI -1 to + 1 mOsmol/kgH2O; *P* = 0.008) compared with 40 min. The comparison between sexes revealed greater plasma osmolality in males at 5, 10, 20 and 40 min (*P* ≤ 0.038) compared to females.

### Electrolytes

**Fig. 2:**
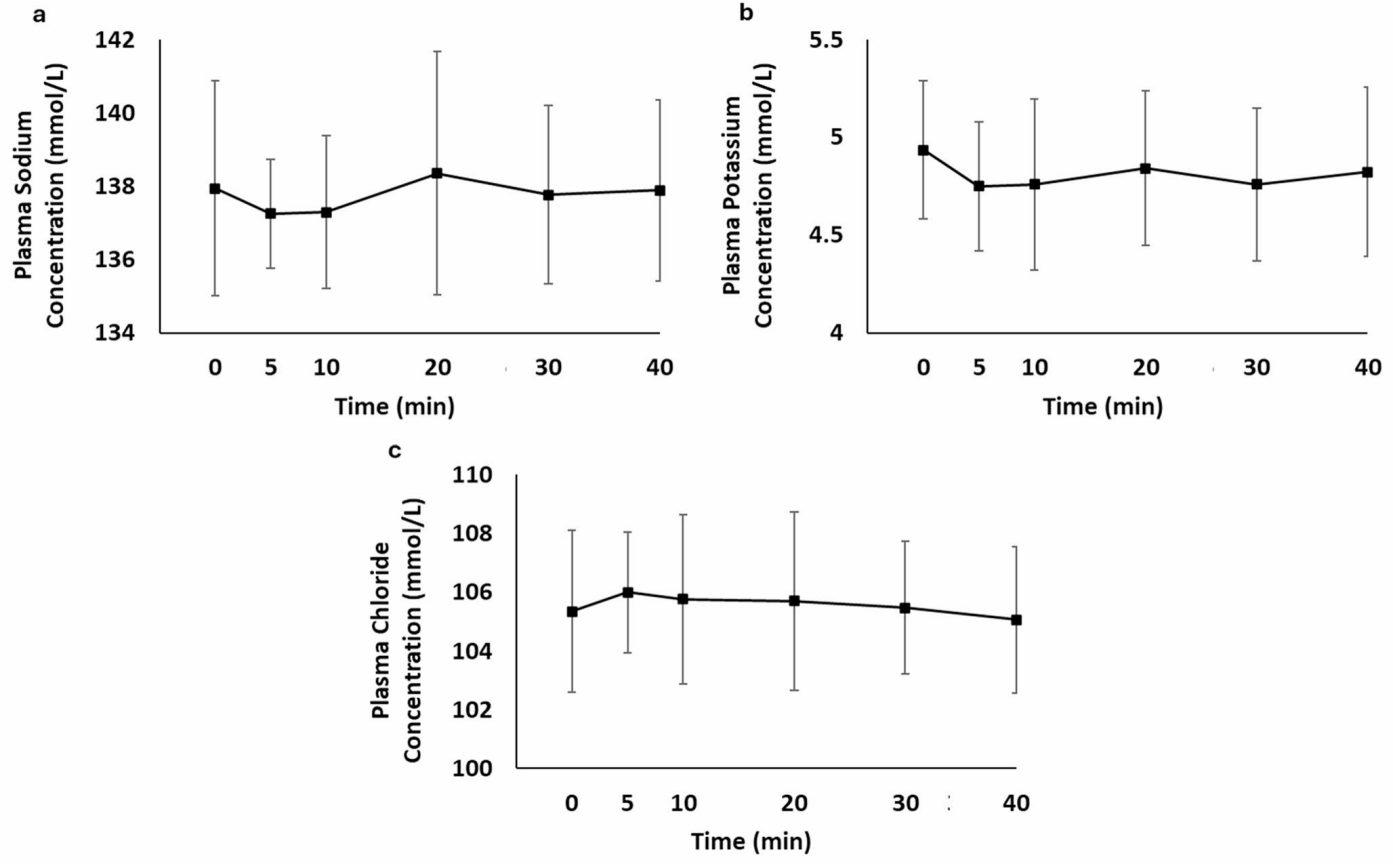
Plasma sodium (mmol/L; **a**), potassium (mmol/L; **b**) and chloride (mmol/L; **c**), immediately upon sitting (0 min) to 40 min. Presented data are mean ± SD

There was a main effect for plasma potassium concentration (*P* = 0.041) but post-hoc tests revealed no differences between 40 min and any other time points (*P* ≥ 0.056; Fig. 2b). There were no main effects for plasma sodium (*P* = 0.604; Fig. 2a) or chloride (*P* = 0.081; Fig. 2c) concentrations.

## Discussion

This study demonstrated that blood/plasma volume, and plasma osmolality required 10 min of seated rest to stabilise, as indicated by a lack of a significant difference to 40 min, following a change in posture from walking to sitting.

Plasma volume increased ~ 6% across the first 10 min of seated rest (from 0 to 10 min), indicating that haemodynamically stabilised measurements of plasma volume in a seated position require at least 10 min of postural control, on average. For context, substantial fluid manipulation, via exercise-induced dehydration of ~ 3% body mass loss (Funnell et al. 2023) and via 24 h complete fluid restriction (Corney et al. 2015) resulted in ~ 5% and ~ 4% reductions in plasma volume, both less than the change observed with sitting in the present study. This demonstrates that lack of postural control in such studies could mask the detection of important intervention-induced changes in fluid balance. Beyond hydration or environmental physiology research, this level of control may also be warranted where plasma biomarker concentrations are corrected for changes in plasma volume (Zappe et al. 1993; Funnell et al. 2023). The changes in measurement exceeded the analytical coefficient of variation (blood volume CV 0.7% with reported changes of 2–4%; haemoglobin CV 0.7% and haematocrit CV of 0.5% are integrated to calculate plasma volume with reported changes of 2–6%; plasma osmolality CV of 0.3% with reported change of 0.7%), providing confidence that there is a meaningful physiological impact on blood-based biomarkers from postural change. Plasma osmolality is more tightly regulated than plasma volume through fluid shifts in vasculature and interstitium, as well as changes in electrolyte concentrations (Cheuvront and Kenefick 2014); however, a decrease in osmolality was observed upon sitting. Conversely, Shirreffs and Maughan (1994) reported no change in serum osmolality but our analysis suggested the females (3 in Shirreffs and Maughan 1994; 8 in current study) may have driven the significant change in plasma osmolality. However, the mechanism underlying this observed sex difference is unknown. It could be speculated that females may be more sensitive to changes in fluid shifts because of having a lower percentage of extracellular fluid due to body composition differences (females have greater adipose stores relatively to males; Ritz et al. 2008) and body water content (absolute and relative; Wickham et al. 2021) compared to males. Although investigating sex differences were unplanned post-hoc exploratory analyses and should be considered as pilot observations. Thus, the sex difference response and underlying mechanisms should be explored in future studies. Whilst this study found changes in plasma osmolality and blood and plasma volume, a similar change was not found in the electrolyte concentrations which might be due to the smaller changes in electrolytes contributing to the aggregate effect on plasma osmolality or reflect that only three electrolytes were measured.

The findings of this study suggest that, whilst postural control is required for stabile measurements of blood/plasma volume and plasma osmolality, the time required for stabilisation is shorter than previously reported for supine rest (Hagan et al. 1978; Shirreffs and Maughan 1994). This may be explained by the greater change in posture (i.e., lower legs and upper body do not change position for seated vs. standing posture), as well as the greater gravitational force and hydrostatic pressure (Harrison 1985) with supine rest. Therefore, and depending on the study design, using upright seated rest instead of supine rest could decrease the time commitment for participants in research studies, helping ease the burden on participants and improve recruitment. Importantly, we included 20 min low-intensity activity prior to sampling since activity decreases blood and plasma volume further compared to standing (Liddle et al. 2018) and to simulate active transport to blood collection sites. Therefore, this gives confidence that we captured the largest shift in plasma volume upon assuming a seated position, meaning in settings where less activity is performed, we would expect the measured variables to stabilise in a similar or lesser time. However, future research should explore whether longer postural control prior to blood sampling is required after moderate and high-intensity exercise. The picture could be further complicated when considering postural stability and the consequences of high intensity exercise which may diminish after 10 min of rest (e.g. plasma lactate concentrations). Another consideration would be the role of skeletal muscle pump activation on walking and the effect on blood volume and subsequent blood marker measurements. Duration, speed of walking, incline and the timing in relation to sample would require careful investigation. Furthermore, whilst there were no statistical differences from 10 min onwards, there was some degree of inter-individual variation in stabilisation responses (supplementary Fig. 1). Therefore, future studies should seek to understand the intra-individual variation in biomarker stabilisation time to help optimise blood sampling protocols and reduce the potential influence of postural factors.

## Conclusion

Ten minutes of upright sitting following light walking (i.e., simulated active travel) appears sufficient for blood and plasma volume, and plasma osmolality to stabilise on average. The implication of blood sampling without postural stability for hydration markers is that measurements may not give valid data, potentially exaggerating or missing important physiological responses. Future research should consider intra-individual and sex-based responses, as well as whether postural stabilisation is required for measurement of other commonly measured biomarkers.

## Supplementary Information

Below is the link to the electronic supplementary material.

Supplementary Material 1

## Data Availability

Data are available from the corresponding author upon reasonable request.

## Abbreviations

ANOVA: Analysis of variance
CI: Confidence interval
CV: Coefficient of variation
EDTA: Ethylenediaminetetraacetic acid
IQR: Interquartile range
SD: Standard deviation
