# Supplementary material for: At least 10 minutes of seated rest is required for stabilisation of blood-based hydration biomarkers
Source: Eur J Appl Physiol. 2025 Nov 21;126(4):2155–60. doi: 10.1007/s00421-025-06048-x (PMC13171926; doi:10.1007/s00421-025-06048-x)

**Supplementary figure 1.** Individual participant data for blood volume change (%; a), plasma volume change (%; b), plasma osmolality (mOsmol/kgH_2_O; c), immediately upon sitting (0 min) to 40 min.


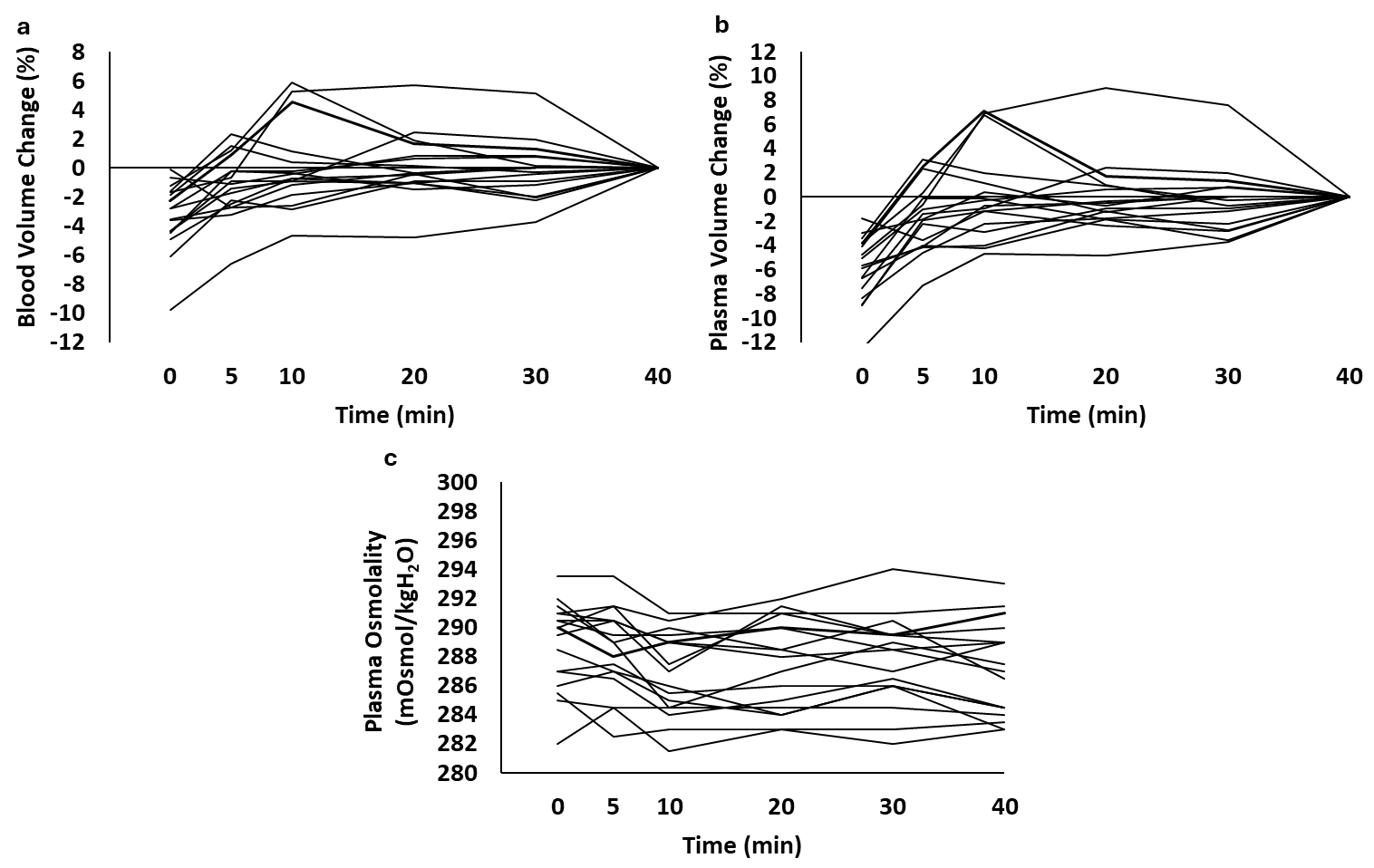

Supplement: Supplementary file 1 — Supplementary Material 1 [file 421_2025_6048_MOESM1_ESM.docx]
